# Transformative dimensions of lifelong learning: Mezirow, Rorty and COVID-19

**DOI:** 10.1007/s11159-020-09859-6

**Published:** 2020-09-25

**Authors:** Saskia Eschenbacher, Ted Fleming

**Affiliations:** 1 Akkon University of Applied Human Sciences, Berlin, Germany; 2 Teachers College, Columbia University, New York, USA

**Keywords:** transformation theory, Rorty, Habermas, disorienting dilemmas

## Abstract

COVID-19 has done significant damage to individuals, families, workers and the economy. What is not known about the virus is part of the problem, and the knowledge gap drives an unprecedented and urgent search for knowledge. This article explores the challenges for lifelong learning and the relevance of transformative learning. Disorientation, *disorienting dilemmas* and *critical reflection* are the ingredients of such learning, since we can only *learn* our way out of this situation. The authors present American adult educator Jack Mezirow’s theory of *transformative learning* (TL) as an appropriate learning framework for lifelong learning. They draw on the work of American philosopher Richard Rorty and German philosopher and sociologist Jürgen Habermas to re-shape TL so that it supports the kind of learning that is sufficiently complex and nuanced to enable us to deal with contradictions, ambivalence and meaning-making in a world where *not-knowing* is the new normal.

## Résumé

Les dimensions transformatrices de l’apprentissage tout au long de la vie : Mezirow, Rorty et la COVID-19 – La COVID-19 a causé des dommages considérables aux individus, aux familles, aux travailleurs et à l’économie. Ce que nous ignorons du virus fait partie du problème et cette absence de savoir impulse une quête de connaissances urgente et sans précédent. Cet article se penche sur les défis qui se posent à l’apprentissage tout au long de la vie et sur la pertinence de l’apprentissage transformateur. La désorientation, les *dilemmes désorientants* et la *réflexion critique* sont les ingrédients de cet apprentissage étant donné que nous ne pouvons sortir de cette situation qu’en *apprenant*. Les auteurs présentent la théorie de *l’apprentissage transformateur* du professeur américain en éducation des adultes Jack Mezirow comme un cadre didactique approprié pour l’apprentissage tout au long de la vie. Ils s’inspirent des travaux du philosophe américain Richard Rorty et du philosophe et sociologue allemand Jürgen Habermas pour refondre l’apprentissage transformateur de sorte qu’il accompagne un type d’apprentissage suffisamment complexe et nuancé qui nous permette de faire face aux contradictions, à l’ambivalence et la recherche de sens dans un monde où *ne pas savoir* est la nouvelle norme.

## Introduction

Suddenly, everything has changed. The challenges of the COVID-19 pandemic go far beyond those which medical and economic experts are facing. There are unimaginable tragedies for families and disaster for those who have lost or will lose their jobs. Medical staff and experts work to make sense of what they have to do for their patients, and pharmacological researchers work to find therapeutic solutions. Citizens struggle with existential threats to their lives, they search for meaning and for ways to manage their own risks. Social solidarity and physical distance are a continuing requirement as everyone is at once part of the problem and part of the solution.

In the midst of the global crisis of the First World War, American philosopher, psychologist and educational reformer John Dewey wrote *Democracy and Education* (Dewey 1916). He resisted any thinking that he believed intended merely to console, isolate or narrow the mind and wrote passionately about an education that would open minds to new concepts and ideas that would respond to the demands of actual human experience and of society. His understanding that moral living is about doing what the known demands seems particularly appropriate in the face of the current crisis. The ability to question, to reflect, to grow, to converse and to learn are key Dewey concepts. His spirit of enquiry is a central characteristic of real democracy.

Educators are experiencing disorientation, questioning previously held assumptions and seeking adequate pedagogical responses to meet learning needs. Robert Kegan and Lisa Lahey (2009) understand *learning needs* as located in a context of increasing complexity both in terms of the level of knowledge available and in terms of the mental systems required to deal with more complex knowledge. They frame this as a developmental task for adults – in much the same way that Dewey saw growth as the aim of education (Dewey 1916). Like never before we are required to understand, respond to and act with sophisticated and expert knowledge. This is knowledge about infections, immunity, resistance, vulnerability, vaccines and knowledge from microbiology, epidemiology and social theory with a mindset created in a pre-pandemic era. What ordinary citizens know is overshadowed by what is not known and yet needs to be known. However, as learners and teachers we are compelled to make sense and meaning of what could be called an absurd situation. Workers from the lower levels of socio-economic scales have become essential workers, while at the same time they are members of high-risk groups in society. Resistance, competing economic interests, confusion and prejudice may conspire to undermine recovery. Broader social issues will certainly have consequences including migration and important international commitments to achieving the United Nations Sustainable Development Goals (SDGs) and implementing lifelong learning (English and Mayo 2019).

In *The Plague* (Camus 1960), originally published in 1947, we see the imagined search for clarity unfold in the midst of a plague, and when it proves unattainable, the result is absurdity. The absurdity is in the tension between not knowing what we want to know and refusing to give way to nihilism. What is left is rebellion, to struggle to make sense and impose form. The plague in the novel represents indifference rather than heroic vigilance of the kind required to combat the plague. In Oran (northwest Algeria), where this plague rages, there are townspeople and volunteers. The townspeople are passive and victims, who fight and lash out. Volunteers form sanitary squads and bury the dead (ibid., p. 131). The doctor, Rieux, also struggles – because it is his job. Both he and the volunteers talk of love and decency. They show compassion, move beyond the sleep of victims and survive – out of a sense of common decency and doing their job (ibid., p. 136). “And thus I came to understand that I, anyhow, had had plague through all those long years” (ibid., p. 205). “[O]n this earth there are pestilences and there are victims, and it’s up to us, so far as possible, not to join forces with the pestilences” (ibid., p. 207). In our current situation, we might identify with the struggle against the plague within, the inertia of unquestioned assumptions – and the way forward is to search for meaning, for growth, for transformation.

In this article we present the theory of *transformative learning* (TL) as a way forward and expand on its possibilities in the context of COVID-19 by linking concepts from Jürgen Habermas and Richard Rorty in order to bring current educational insights to bear on tackling the crisis rather than join forces with the pestilences.

## Learning *transformatively* in the light of COVID-19

As learners and educators we are among the “not knowing”, searching for pathways to foster what is key to the work of Dewey (1916), namely the ability to question, reflect, converse, grow and learn. Guided by what learners and society need in these times, the theory of transformative learning (TL) opens up possibilities to meet these requirements. Transformative learning explains how we make meaning, interpret experiences, and how we question, reflect on and converse about these experiences in order to develop and grow. It is

an approach to teaching based on promoting change, where educators challenge learners to critically question and assess the integrity of their deeply held assumptions about how they relate to the world around them (Mezirow and Taylor 2009, p. xi).

This theory is concerned with assisting learners to explore and enter into a critical dialogue with their guiding assumptions. Transformative learning is the struggle with, and transformation of, unquestioned assumptions. Challenging taken-for-granted *meaning perspectives* (Mezirow 1991) and searching for meaning, growth and transformation offer a way forward as TL theory suggests – and so enables one to emancipate oneself from values and meanings one has uncritically assimilated. The opportunity to learn transformatively arises out of the experience of crisis or disorientation. In the light of COVID-19, pre-pandemic mindsets are dysfunctional.

*When our meaning perspectives are questioned, the coherence-producing mechanism of our minds is interrupted.* We are no longer able to interpret the situation based on our previous experiences (Mälkki 2019, p. 64, emphasis in original).

American adult educator Jack Mezirow (1991, pp. 168–169) outlines ten phases within the process of *perspective transformation,* starting with (1) a *disorienting dilemma,* which sets the stage for (2) an exploration of feelings like guilt or shame that arise in the wake of the crisis or dilemma. In a third step, (3), learners critically assess and reflect on their guiding assumptions underlying their current meaning perspective. What follows is (4) the realisation that one’s personal problem is shared and (sometimes) a public issue: the public breaks into the private sphere and learners realise that others have negotiated and undergone similar changes and challenges. In the next phase, (5), learners explore alternative ways of being and living in terms of relationships, roles and actions. This phase is complemented by another phase, where (6) learners plan (new) courses of action and (7) acquire new knowledge in order to put these courses of action into practice. In the aftermath of (8) learners trying out these new roles (provisionally), they (9) build (self-) confidence and competence and (10) re-integrate into their lives, employing a new, transformed (meaning) perspective.

The experience of *not-knowing*, or the challenge of combining social solidarity with physical isolation provide the kind of disruptions that TL theory defines as *disorienting dilemmas* (Mezirow 1991). Feeling ashamed of being disoriented might be accompanied by fear, loss and (anticipatory) grief, not knowing how to cope with the current crisis. Hitherto unquestioned assumptions about freedom of movement, social solidarity and the limits of knowing become fragile. Existential uncertainty is both a global crisis and an individual experience – also for learners. How does one sustain relationships while observing physical distance?

How can we live in the *not-knowing*, cope with uncertainty, ambiguity and alienation? A learning process that is an “epiphanic, or apocalyptic, cognitive event – a shift in the tectonic plates of one’s assumptive clusters” (Brookfield 2000, p. 139) involves a fundamental reordering and redescription of how one thinks, feels or acts. Thus, TL becomes not only a possibility but a necessity.

What gets transformed in TL? According to Mezirow, what gets transformed is what he terms a *frame of reference* or a *meaning perspective,*

the structure of assumptions and expectations through which we filter sense impressions. It involves cognitive, affective, and conative dimensions. It selectively shapes and delimits perception, cognition, feelings, and disposition by predisposing our intentions, expectations, and purposes. It provides the context for making meaning (Mezirow 2012, p. 82).

Mezirow’s *perspective transformation* (Mezirow 1978b) highlights the necessity to create a critical awareness of how perspectives and guiding assumptions limit our ways of living and being in the world.

Engaging in a *critically reflective* (Mezirow 1991) conversation about assumptions opens the possibility of transforming them, so that learners’ assumptive clusters provide a framework where they gain an increased ability to cope with ambiguity, uncertainty and contingency as their clusters become more inclusive, discriminating and open to further change. Transforming core meaning structures allows learners to make sense of new experiences that are challenging. According to Mezirow, *premise reflection* is

the dynamic by which our belief systems – meaning perspectives – become transformed. Premise reflection leads to more fully developed meaning perspectives, that is, meaning perspectives that are more inclusive, discriminating, permeable (open), and integrative of experience (Mezirow 1991, p. 111).

We know for sure that this premise reflection has to address both individual and societal/global dimensions, since the current crisis is simultaneously experienced as an individual and a global crisis.

The theory of TL addresses a type of learning where the individual and the social intersect (Fleming 2002). Mezirow (1978a) always described the *transformation learner* as one who is aware of how the public breaks into the private sphere and understands that these spheres are connected. The choice of social action resides with the learner, as collective and social transformation may be separate entities from individual transformation (Mezirow 1989). A perspective transformation happens within the learner, and

the site of change – as well as agency – is envisaged primarily in terms of the transformation of the inner mental landscape of an individual learner which may, or may not, have broader social consequences (Finnegan 2019, p. 48).

Fergal Finnegan (ibid.) argues that TL is not an individualistic theory, as Mezirow puts an emphasis on intersubjective learning through discourse. Intersubjective learning refers to any learning that happens between people who communicate in order to support or understand each other. Group discussion is a good example.

In broadening current perspectives, TL helps us live with uncertainty and ambiguity, and fosters a democratic learning culture. Mezirow identifies the need to cope with ambiguity as central to TL, “identifying the common in the contradictory, tolerating the anxiety implicit in paradox, searching for synthesis, and reframing” (Mezirow 2012, p. 80). Adults need to be able to hold positions that may seem to conflict or contradict each other and to deal with more complexity, and struggle less with contradiction and opposites (Kegan 2000).

However, learning transformatively is

not a continuously joyful exercise in creative self-actualisation. It is psychologically and politically dangerous, involving risks to one’s livelihood, social networks, and psychological stability (Brookfield 1990, p. 179).

Interestingly, Stephen Brookfield mentions several layers of transformative learning here, all of which are reflected in the current crisis: psychological stability is put at risk as *edge-emotions* (Mälkki 2011)^1^ that arise are experienced individually; social networks are at stake as solidarity has to be lived through physical distancing. The experience of being physically distant while being connected through social media adds additional challenges. Transformative learning is also politically dangerous, as this crisis reveals structural injustices. Risks are not equally distributed, and frontline workers carry a heavy burden on behalf of everyone. Social inequalities are amplified. Reopening the economy raises questions about who may be carrying the risks. Transformative learning is also empowering.

Transformative learning begins with a disorienting dilemma: an individual is unable to make sense of an experience within her or his current pre-pandemic frame of reference; adapting new learning may no longer suffice.

When a meaning perspective can no longer comfortably deal with anomalies in a new situation, a transformation may commence. Adding knowledge, skills, or increasing competencies within the present perspective is no longer functional; creative integration of new experiences into one’s frame of reference no longer resolves the conflict (Mezirow 1978b, p. 104).

So how can we think of education as *leading out* of our *not-knowing* in this age of COVID-19? How can we assist learners to find ways through the current crisis? What do learners need in their search for meaning, growth and transformation?

Learners who are experiencing a disorienting dilemma, originating from their having lost their way in the world, feel the urgency to rediscover a sense of direction; they are in need of “an exploratory, associative, open-ended, tolerant exchange of intimations free from the demand that it [should] issue in conclusions binding on all” (Arcilla 1995, p. 7). This sense of direction needs to be rediscovered in the light of disorientation, when notions of health or normality are disrupted.

In an interview on the experience of grief arising from COVID-19, writer and grieving expert David Kessler argues that the experience “breaks our sense of safety. We’re feeling that loss of safety” (Berinato 2020, p. 1). The disorienting dimension arises from collective experiences of losing our sense of general safety as a “loss of normalcy; the fear of economic toll; the loss of connection” leads to grieving collectively (ibid., p. 1). We are experiencing what Kessler terms *anticipatory grief*, “that feeling we get about what the future holds when we’re uncertain” (ibid., p. 1). One might not always be fully aware of these edge-emotions (including grief) or the character of the emotions arising. *Edge-emotions* “offer us access to knowledge that we could not perceive from our existing meaning perspectives” (Mälkki 2019, p. 69). Even though people are trying to get back to their comfort zones, to escape these edge-emotions and return to the previously known “normal”, the individual and global crisis arising from the COVID-19 pandemic does not seem to offer us a way back to this known “normal”. Kaisu Mälkki argues that in order to learn transformatively, we need to develop our ability to embrace edge-emotions, which come into play when we are challenging the integrity of deeply held assumptions. Following Mälkki’s approach, the way forward is to reframe these edge-emotions as an invitation to explore our assumptions in the light of the current crisis. Kessler’s advice is similar to Mälkki’s suggestion that we acknowledge what we are going through so that we can find or regain some control in acceptance (Berinato 2020).

## Thoughts from a Habermas perspective

Mezirow (1991) draws heavily on the work of German philosopher and sociologist Jürgen Habermas (1971, 1984, 1987) and builds on the Habermasian ideas of *discourse, instrumental, communicative* and *emancipatory knowledge,* and the role of argument and rationality as key concepts in his TL theory. An understanding of *participatory democracy* that rests on *critical reflection* of originally unproblematic assumptions is central to TL. Mezirow locates a Habermasian notion of *discourse* as the process through which one learns transformatively.

Learning is central for Habermas, and in his view not *learning*, but *not learning* is the phenomenon that calls for explanation (Habermas 1975, p. 15).

It is not surprising that Habermas relates adult learning to his vision of a democratic society. He refers to this relation as the *adult learning project* (Habermas 1987) and associates democracy with free and unrestrained communication. Habermas links “the importance of learning how to reason to adults’ ability to participate in democratic decision making” (Brookfield 2005, p. 1131). Habermas (1987) postulates an adult learning crisis in modern society, arguing that adults are not sufficiently prepared for what is central to his vision of a democratic society, namely participation in public discourse.^2^ Habermas’ view of what adults need to be active citizens departs from the uncritical version of lifelong learning, where a lack of basic skills in concert with employability is central for adults in order to fulfil their roles as active citizens participating in democracy. Maren Elfert (2018) refers to the way lifelong learning has been emaciated and today bears little resemblance to its original meanings. She asserts that, in the European Union (EU), “Lifelong learning began as a radical idea with a strong political dimension, which asked questions about justice and equality, the distribution of resources and the exercise of power” (Elfert 2018, p. 215). It has instead become

de-politicized and “transformed” to make it fit into the agenda of the marketplace, turning it into a euphemistic label for a neoliberal worldview, in which the individual is held responsible to invest in her human capital, in the name of a false notion of freedom (ibid.).

Ever since a number of policymaking entities and other international institutions such as the Organisation for Economic Co-operation and Development (OECD) and the EU have made lifelong learning a policy priority and an instrument for supporting economic development there has been a conflict about the precise meaning of the term *lifelong learning.* The original concept of *lifelong education* supported by the United Nations Educational, Scientific and Cultural Organization (UNESCO) in the 1960s emerged from an “egalitarian and democratic spirit inherent in the idea of education as a human right” (Elfert 2019, p. 540). It

reached its fullest expression in UNESCO’s work on the concept of lifelong learning, represented by two publications, namely *Learning to be* … (Faure et al. 1972) and *Learning: The Treasure Within* … (Delors et al. 1996) (Elfert 2019, p. 540).

But this spirit has changed:

UNESCO’s utopian and citizenship-oriented vision of lifelong learning has largely been supplanted by more economics-driven proposals for education put forward by other international organisations (ibid., p. 540).

Humanistic (Wain 2001), radical (Coffield 1999) and critical theory-inspired ideas (Negt 2008) continue to stimulate the debates.

In a recent interview, Habermas describes our current situation as one we have never experienced before, namely having so much knowledge about our lack of knowledge and being forced to act and live under the uncertainty of *not-knowing* what we need to know. This reflects a global and learning crisis and simultaneously an individual experience. The social, political or economic consequences are not clear (Schwering 2020).

Habermas, as Brookfield points out, is aware of the importance of adults’ ability to deal with the struggle of contradictions and tensions inherent in democracy:

People need to experience the contradictions and tensions of democracy and to learn how to navigate through these while also learning the uncomfortable ontological truth that they are often unnavigable (Brookfield 2005, p. 1164).

From his youth Habermas was convinced that democracy was the path to the future in which there would be a “denser and more fragile network of relationships built on reciprocal recognition” (Habermas 2008, p. 17). It is at this point that Mezirow’s (1991) TL theory, Kegan and Lahey’s (2009) developmental approach and Habermas’ (1975) notion of learning democracy intersect: They all see a need to help adults learn to live with ambiguity, contingency – and, in the shadow of COVID-19, – one might add uncertainty and *not-knowing*. Finnegan points to Mezirow’s interest in supporting a democratic learning culture, as he

could not be clearer that he is interested in supporting democratic movements and progressive social change – but they are not foregrounded in a systematic way (Finnegan 2019, p. 47).

What we present below is a twofold agenda (Mezirow 1991), where the *lifeworld*^3^ has to be strengthened through democratic movements against the system’s colonisation as well as supporting a critical approach towards the system through learning, reflection and discourse. The lifeworld is not only to be strengthened, but *transformed* in the context of TL theory. This is close to what Mezirow conceptualises as a *frame of reference*, “as having cultural, social and personality dimensions, as the lifeworld” (Fleming 2002, p. 7). It is the philosophical grounding of TL in Habermas’ works that adds the *social dimension* to TL (ibid.). When others serve as *critical mirrors* (Brookfield 2000) in discourse, they enable us to critique our assumptions. *Critical reflection,* according to Mezirow (1991), is situated in a community established by reflective, rational dialogue and is intersubjective by nature – with ties of mutual recognition.

Mezirow (1991, pp. 64–98) relies on Habermas’ notion of *rational discourse* (Habermas 1971, 1984, 1987) to promote TL by exchanging arguments in an environment free from coercion. Learning transformatively involves rediscovering a sense of self-direction insofar as one learns to act on one’s freely chosen values, feelings and purposes instead of those “uncritically assimilated from others” (Mezirow 2012, p. 75). In order to do this, according to Mezirow, one needs to engage in rational discourse by exchanging arguments. Brookfield suggests:

A separation from immediate experience allows adults to reflect back on this experience – usually in conversation with others – in a way, and with a critical edge, that is difficult in daily life. This is the essence of adult critical reflection (Brookfield 2005, p. 1165).

This notion of *discourse* belongs to the public sphere, not to the private sphere (Rorty 1989). As Rorty asserts “Habermas is a liberal who is unwilling to be an ironist” (ibid., p. 61). Being primarily interested in democracy and the public sphere, Habermas is less concerned with (ironist) projects of self-fulfilment or self-development, which belong to the private sphere. Therefore, TL theory, as much as it focuses on individuals experiencing a *perspective transformation,* is mainly concerned with a discursive format to promote the kind of TL which is appropriate for deliberative decision-making processes and participation in democracy. What is missing is a philosophical grounding that reflects both processes of transformation and related, suitable concepts to foster TL on a meta-theoretical level (Eschenbacher 2019). In the context of COVID-19, this becomes apparent as a dilemma that needs to be adequately addressed through the theory of TL. One possible way of tackling this is to engage Mezirow in a critical conversation with Rorty.

## Thoughts from Rorty’s perspective

American philosopher Richard Rorty (1989) differentiates the question of how should *I* live *my* life, where no consensus is necessary, from the question of how should *we* live *our* lives, where there is a need for consensus and solidarity. The latter question shares much of what we find in the Habermasian foundation of the theory of TL. Rorty’s work is particularly relevant here, since his concept of *irony* (ibid.) values ideas of freedom and change, or, more precisely, transformation. He embraces an ethic of invention and seeks to open up possibilities to transform our given selves by redescribing our current selves. Transformation can be conceptualised as a *redescription* of ourselves, our situation and our being in the world, whereas Rorty’s idea of *vocabularies* is closer to what Mezirow understands as *frame of reference* (Eschenbacher 2019).

Mezirow’s understanding of *premise reflection* is close to Rorty’s notion of *redescription*. By redescribing the circumstances of our assumptions, we are able to deconstruct them and change our ways of posing and solving problems. We are able to free ourselves from self-imposed limits through the constant process of redescription (Eschenbacher 2019). It is central to Rorty’s ideas that one is not trapped by one way of looking at the world that is forced on us. Our ability to redescribe ourselves and create new *vocabularies* gives us opportunities to transform assumptive clusters, “we create our own selves by redescribing our given selves” (Arcilla 1995, p. 93, with reference to Rorty).

Whenever we encounter the experience of a crisis or dilemma, the limits of our current vocabulary (frame of reference) are being revealed. The integrity of our deeply held assumptions is challenged, and we are invited to ask “Does our use of these words get in the way of our use of those other words?” (Rorty 1989, p. 12) when we experience the limits of describing our current situation. For Rorty, the “ability to appreciate the power of redescribing, the power of language to make new and different things possible and important” is key to the idea that there is no such thing as “The One Right Description” (ibid., pp. 39–40). There are alternative descriptions that allow us to expand our repertoire or broaden our current meaning perspectives.

René V. Arcilla reads Rorty’s work from an educational perspective, arguing that

it paves the way for a promotion of ironic self-formation and compassionate communal solidarity as the two principal aims of an education that would support a liberal culture (Arcilla 1993, p. 203).

Kenneth Wain in turn has identified Arcilla as an insightful interpreter of Rorty’s “new hope for education in the light of ‘the crisis in modernity’” (Wain 1995, p. 395). In Rorty’s work – and in TL theory – both spheres, the private and the public, are discussed. However, unlike TL theory, Rorty identifies the usefulness of exchanging arguments in the quest of transformation:

“[t]he vocabulary of self-creation is necessarily private, unshared, unsuited to argument. The vocabulary of justice is necessarily public and shared, a medium for argumentative exchange” (Rorty 1989, p. xiv).

Rorty’s distinction between private and public *vocabularies* adds a valuable dimension to the theory of TL. By placing Habermas’ notion of *discourse* at the centre of Mezirow’s theory, TL theory lacks the kind of dialogue that is more suitable to fostering *perspective transformation* within the personal sphere. Even though TL theory focuses on the individual without being an individualistic theory of adult development, it lacks a form of dialogue that is not merely about exchanging arguments and the non-coercive force of the better argument. When adult learners are searching for answers to the question of how to live one’s life, it is not so much about finding “the” objective truth, one correct way to live life, or arriving at a consensus with others. Instead, it is more about the realisation that there are always other possibilities to explore, that one is not trapped by one way of looking at the world and one way of living.

By bringing Mezirow’s theory of TL into dialogue with Rorty’s work, TL theory strengthens its potential to support individuals in coping with challenges in their private lives. It adds a valuable dimension to the discourse of *lifelong learning* which is so often reduced to being concerned with people’s employability and their capacity to be or become active citizens. Mezirow created a theory that is less concerned with people’s employability – or, to use Habermas’ words – an *instrumental approach* (Habermas 1987). On the other hand, the idea of personal development or individual fulfilment is less prominent within the lifelong learning discourse. The experience of an existential crisis – like COVID-19 – reveals the absence of a debate about the idea of personal development and an ability to cope with crises apart from the notion of “employability” within the lifelong learning discourse. By engaging in a dialogue between Rorty and Mezirow, this gap can not only be closed within TL theory, but also addressed within the lifelong learning discourse.

Educators need to create some kind of safe (enough) space where learners can work their (individual) ways through experiencing existential crises accompanied by *edge-emotions* (Mälkki 2011, 2019). Reflective critical *discourse* of the kind Mezirow (1991) recommends might not be the most adequate format to create a safe (enough) space. Instead of looking for the coercive power of the better argument and arriving at a tentative consensus on how to deal with the consequences and with edge-emotions, the theory of TL needs to be broadened. There is no need for consensus regarding the way in which one copes with COVID-19 as an individual, or how to live one’s life. There is no “One Right” way to do this. We need to foster new kinds of dialogue suitable for supporting learners in transforming their individually experienced crises into learning opportunities – *limit situations* (Freire 1972).^4^ However, there is also a need for public discourse, for a consensus on how *we* want to live *our* lives together in the light of COVID-19, how to live in solidarity and how to foster democratic learning processes.

As (adult) educators, we can invite learners to enter into a conversation with their emotions, to work *with* them instead of avoiding them. The current crisis, being experienced both globally and individually, urges people to learn transformatively. It also creates a learning opportunity to engage in the kinds of conversations that are supportive to coping with these challenges either in an individual way or as a society. Since these different ways of coping belong to different spheres (the private and the public, respectively), there is a need to address both spheres in ways that are adequate for each.

Arcilla (1995) suggests the concept of *conversational edification* as “*the power to converse reasonably with others for the purpose of edifying oneself*” (Arcilla 1995, p. 105, emphasis in original). His concept is especially helpful for learners experiencing disruptions to originally unproblematic ways of being and living:

As we edify ourselves in response to events that befall us … we develop our ability to weave contingent but consistent stories of the course of our own lives (ibid., p. 100).

This calls for a format which is different from discourse and argumentation, one that allows for an exchange and for exploration of different vocabularies without the necessity of arriving at a consensus or a final *vocabulary* in Rorty’s terms.

Rorty’s (1989) *vocabularies* comprise more than just the ways in which we describe ourselves and the world we live in. Instead of asking what the correct way of looking at the world is, and discussing arguments to see what we all can agree on to be true, the alternative, namely exploring different vocabularies as *proposals* regarding how one *might* live can be understood as invitations to leave the (mental) homes we have hitherto occupied. Rorty’s *vocabularies* provide the ground for justifying actions, beliefs and living one’s life. They are the words with which we express

our deepest self-doubts and our highest hopes. They are the words in which we tell, sometimes prospectively and sometimes retrospectively, the story of our lives (Rorty 1989, p. 73).

Like Mezirow, Rorty wants “to liberate us from the dead weight of past vocabularies and open up space for the imaginative creation of new vocabularies” (Bernstein 2016, p. 52).

Rorty’s (1989) concept of *irony* offers an attitude that doubts our own vocabulary in a radical and ongoing way. It is through the experience of (existential) crisis that learners become aware of the limits of their vocabulary in use. Instead of trying to arrive at a *final* vocabulary, one we take for granted, Rorty suggests – as does Mezirow – to remain open to alternative vocabularies, to understand them as *tentative*, and *contingent*; as one way of being or one possible vocabulary among others (Eschenbacher 2019). Rorty’s concept of *irony* stresses the idea that we are free to create new vocabularies, to redescribe ourselves and to transform our guiding assumptions. For Rorty (1989), radical and continuing doubts about the vocabulary we employ is one defining criterion for being an *ironist*. Another is the awareness that our most central beliefs are subject to change. “Contingency stresses freedom by keeping open the possibility of metaphorical redescription” (Arcilla 1993, p. 202).

This is also the essence of TL theory: the purpose of transformative learning is to help adults transform their taken-for-granted assumptions about themselves, as well as how they relate to the world. One way forward in coping with feelings of loss, grief and *not-knowing* is to accept them, as Kessler suggests (Berinato 2020). Providing adults with a type of *edifying conversation* that serves as a space where they can redescribe themselves and rediscover a sense of self-direction in order to be better able to cope with COVID-19 is a pathway to the kind of transformative learning that allows learners to broaden their current meaning perspectives, to develop – and ultimately to grow.

## Conclusion

In the light of COVID-19 one might begin to ask what *employability* and *active citizenship,* as the two central components of lifelong learning (EC 2000), might mean. What if we redescribe the circumstances of lifelong learning so that employability and active citizenship will not remain the only goals of lifelong learning?

In the aftermath of a crisis that is simultaneously an individual and a global experience, what might be worth exploring for those who engage in the discourse on lifelong learning are solidarity and the ability to cope and transform oneself in order to be better able to deal with uncertainty, ambivalence and loss. Ethical dilemmas are far from solved or adequately addressed when we continue to engage in discourses about how to increase employability and improve economic outcomes. Instead, it might be interesting to explore different options about how to move from unjust to more just and caring societies; how we can co-create societies and a world in which we can *all* live, and not join forces with the pestilences. This endeavour will involve redefining the relationship between work and lifelong learning.

Christine Hof (2017) argues that TL theory may add an important dimension to the lifelong learning discourse, since it enhances our understanding of learning throughout the life course. The experience of *not-knowing*, of lacking knowledge to cope with situations provoked by a crisis, can be seen as a possible point of entry for learners to reflect on current ways of knowing and being in the world, and to engage in changing these ways.

Transformative learning, in conversation with Rorty, adds an additional, hitherto overlooked dimension to the discourse on lifelong learning, namely the project of *personal development*. This personal project of *edification* needs to be added to the discourse on lifelong learning in the sense of being better able to live with contingency, uncertainty and ambiguity. This may help us master the challenges and demands of adults’ everyday lives even in the face of existential crises such as the one we are currently experiencing. While all of these aspects have become a prerequisite for mastering adult life, they have not yet been given enough consideration in the course of one’s lifespan from the perspective of lifelong learning. Transformative learning adds this insight to the discourse. According to both Habermas and Mezirow, participatory, active citizenship might be less a matter of employability than the current discourse on lifelong learning suggests. To foster communicative learning and reason as an *adult learning project*, and to establish a global dialogue on how *we* want to live *our* lives, might provide an avenue worth exploring in the face of this current existential crisis.

Transformative learning (TL) adds a critical dimension to lifelong learning’s discourse on private projects of self-development, namely being better able to cope with existential individual crises on the one hand, and active citizenship on the other. This becomes apparent in the light of COVID-19. Transformative learning also has the potential to transform a global and individual crisis into a learning experience which addresses both the individual and society. Though the plague may be within and without, the learning challenge is to remain wide awake and not succumb to the pestilences and become victims.

## Footnotes

1 In her development of edge-emotion theory, Kaisu Mälkki explains that while “*comfort zone* refers to the pleasant experience of being able to make meaning unproblematically within the meaning perspective and maintain the intactness of it; *edge-emotions* refers to the unpleasant feelings that emerge when we are unable to understand or when our meaning perspective becomes questioned. In other words, in these situations we are out of our comfort zone or at the edges of it” (Mälkki 2011, p. 30, italics in original).

2 Traditionally critical theorists of the Frankfurt School hold that “Democracy is the only politically conceived social order that has to be learned, over and over, every day, into old age” as a “process of education and learning” (Kluge and Negt 2014, p. 452).

3 The lifeworld is a pool of intuitive knowledge about the objective, social and intersubjective world inhabited by people. It is employed, usually without thinking, in order to establish and sustain interactions. According to Mezirow it is “a vast inventory of unquestioned assumptions and shared cultural convictions, including codes, norms, roles, social practices, psychological patterns of dealing with others and individual skills” (Mezirow 1991, p. 69).

4 People are limited by their experience, social situations and abilities as to what they can achieve. Limit situations are a product of history, made by people (with vested interests) and are not permanent. The existence of these limiting situations are, according to Brazilian educator and philosopher Paulo Freire, opportunities for making progress by overcoming them (Freire 1972). They are not the “impossible boundaries where possibilities end, but the real boundaries where all possibilities begin” (Freire 1972, p. 71).
